# Tear Film Pharmacokinetics and Systemic Absorption Following Topical Administration of 1% Prednisolone Acetate Ophthalmic Suspension in Dogs

**DOI:** 10.3389/fvets.2020.571350

**Published:** 2020-10-27

**Authors:** Lionel Sebbag, Nicolette S. Kirner, Larry W. Wulf, Jonathan P. Mochel

**Affiliations:** ^1^Department of Veterinary Clinical Sciences, College of Veterinary Medicine, Iowa State University, Ames, IA, United States; ^2^Department of Biomedical Sciences, SMART Pharmacology, College of Veterinary Medicine, Iowa State University, Ames, IA, United States; ^3^Lloyd Veterinary Medical Center, College of Veterinary Medicine, Iowa State University, Ames, IA, United States; ^4^PhAST Laboratory, College of Veterinary Medicine, Iowa State University, Ames, IA, United States

**Keywords:** prednisolone acetate, pharmacokinetics, corticosteroid, tear film, systemic absorption, ocular pharmacology, tear collection, canine

## Abstract

The study aimed to determine the tear film pharmacokinetics following topical administration of 1% prednisolone acetate—assessing whether two drops would provide a superior kinetic profile compared to one drop—and to determine the fraction of an eye drop that reaches the systemic circulation in dogs. Two separate experiments were conducted in eight healthy Beagle dogs: (i) Instillation of 1 drop (35 μL) or 2 drops (70 μL) of 1% prednisolone acetate ophthalmic suspension in each eye, followed by tear collections with Schirmer strips from 0 to 720 min; (ii) Instillation of 1 or 2 drops of 1% prednisolone acetate in both eyes 4 times daily for 3 days, followed by blood collection 10–15 min after each topical administration on Day 3. Tear and blood samples were analyzed with high performance liquid chromatography to determine the levels of prodrug (prednisolone acetate), active metabolite (prednisolone) and total prednisolone (prednisolone_total_ = prodrug + active metabolite). Prednisolone levels represented 10 and 72% of prednisolone_total_ concentrations in tears and plasma, respectively, indicating a greater hydrolysis of prodrug in the blood vs. tear compartment. For eyes receiving one or two drops, tear film prednisolone_total_ concentrations were high (~3.1 mg/mL) immediately following topical administration but rapidly decreased by ~45% at 1 min and ~95% at 15 min. No differences were noted between 1 vs. 2 drops in tear film prednisolone_total_ concentrations (including maximal concentration, C_max_) or residual drug levels in tears at any time point (*P* ≥ 0.097); however, instillation of 2 drops provided a higher average tear concentration (C_avg_) and overall drug exposure to the ocular surface (AUC_last_) over the 12-h sampling period (*P* = 0.009). Average plasma prednisolone_total_ concentration represented ≤ 2% of the dose applied to the ocular surface, and did not differ significantly for dogs receiving 1 drop (17 ng/mL) or 2 drops (20 ng/mL) 4 times daily for 3 days (*P* = 0.438). In sum, topical corticotherapy is beneficial for inflammatory conditions of the canine anterior segment given the relatively high concentrations achieved in tears, although caution is warranted to prevent unwanted local or systemic adverse effects.

## Introduction

Topically administered corticosteroid is a common practice in human and veterinary ophthalmology (1, 2). Owing to a wide range of biologic effects, from anti-inflammatory to immunosuppression, topical corticosteroids are indicated for various conditions affecting the ocular surface and the anterior segment of the eye, including dacryocystitis, episcleritis, conjunctivitis, keratitis, and anterior uveitis (1, 2). Multiple formulations of ocular corticosteroids are commercially available, and the choice is often dictated by the properties of the specific drug and associated formulation; in practice, 1% prednisolone acetate is often considered superior to other formulations (e.g., betamethasone phosphate, dexamethasone alcohol, or disodium phosphate) as the acetate derivative of the steroid provides superior penetration in the cornea and anterior chamber (1, 3, 4). However, despite well-documented benefits of topical prednisolone acetate for various ocular disorders, the basic pharmacokinetics (PK) of the drug has yet to be elucidated. This knowledge would help clinicians optimize frequency of administration and better understand the potential toxicity associated with topical corticotherapy at a local and systemic level (1, 2).

Upon instillation on the ocular surface, prednisolone can exert local and systemic effects as follows: (i) mixing with tear film and retention on the ocular surface (tears, conjunctival cul-de-sac); (ii) penetration into the cornea and aqueous humor; and (iii) systemic absorption into conjunctival vessels and nasolacrimal drainage, where the drug can be absorbed by the nasal mucosa or be swallowed (5). As the first step in ocular pharmacology, tear film PK provides important information about the instilled drug (e.g., concentration, precorneal retention time), as well as an indirect and non-invasive method to estimate intraocular drug concentrations, especially for drugs such as prednisolone acetate that readily permeate through the corneal barrier (6). In dogs—a species better suited for translational research in pharmacology than common laboratory animals (7–9)—tear film PK has been described for three ophthalmic drugs to date: fusidic acid (10), ciprofloxacin (11), and oxytetracycline (12). However, direct extrapolation with prednisolone acetate is likely inaccurate as the PK of topically administered medications greatly depends on the physicochemical properties of the formulation (e.g., pH, viscosity, particle size) (13). In particular, prednisolone acetate is a prodrug that requires ester hydrolysis to obtain the active metabolite prednisolone (14); further, prednisolone acetate is a suspension and not a solution, a formulation that could theoretically increase the retention time on the ocular surface as drug particles are sequestered in the conjunctival cul-de-sac (15, 16).

The main purpose of the study was to determine the tear film pharmacokinetics following topical administration of 1% prednisolone acetate in canine eyes, and to assess whether topical administration of 2 drops would provide a superior PK profile compared to a single drop. Prednisolone acetate is a suspension formulation that enhances drug penetration through the cornea, converted into the active metabolite (prednisolone) by esterases following topical administration to the eye (14). Since most of the esterase activity occurs in ocular tissues (e.g., cornea, anterior uvea) and not in tears (17), prednisolone_total_ (prednisolone acetate + prednisolone) was selected as the study's main outcome to provide information about the total amount of drug available for drug diffusion into the eye. Another study objective was to evaluate the plasma drug concentrations when topical 1% prednisolone acetate drops are instilled at a dosage frequency similar to canine patients (i.e., four times daily).

## Materials and Methods

### Animals

Eight Beagle dogs were enrolled in the study. All dogs were neutered (four male, four female), aged 3.0–3.5 years, weighed 7.5–10 kg, and confirmed to be healthy based on a complete physical and ophthalmic examination, including Schirmer tear test-1 (Eye Care Product Manufacturing LLC, Tucson, AZ, United States), tonometry (TonoVet, Icare Finland Oy, Espoo, Finland), slit-lamp biomicroscopy (SL-17; Kowa Company, Ltd., Tokyo, Japan) and indirect ophthalmoscopy (Keeler Vantage; Keeler Instruments, Inc., Broomall, PA, United States). The study was approved by the Institutional Animal Care and Use Committee of Iowa State University (IACUC-18-398), and adhered to the Association for Research in Vision and Ophthalmology (ARVO) statement for Use of Animals in Ophthalmic and Vision Research.

### Tear Film Pharmacokinetics Following Instillation of 1 vs. 2 Drops

In each dog, one eye was chosen at random (Excel software) to receive 35 μL (one drop) of 1% prednisolone acetate ophthalmic suspension (Sandoz Inc., Princeton, NJ, United States), while the other eye received 70 μL (two drops), and this selection was kept constant throughout the study. The experiment extended over 13 consecutive days, with topical prednisolone acetate delivered with a pipette at 8:00 a.m. (each day) and tear fluid collected with dye-free Schirmer strips at the following time points (one collection per day): 0 min (i.e., immediately after instillation and blinking), 1, 3, 5, 15, 30, 45, 60, 90, 120, 240, 480, and 720 min. At completion of tear collection each day, both eyes were thoroughly rinsed with eyewash (Eye Wash, OcuSOFT Inc., Richmond, TX, United States) to avoid any carryover of prednisolone acetate from 1 day to another. For eye drop administration, 1% prednisolone acetate ophthalmic suspension was transferred to Eppendorf vials that were vigorously shaken for 1 min with a vortex mixer (LP vortex mixer, Thermo scientific Inc., Waltham, MA, United States) to homogenize the distribution of drug particles, followed by accurate administration of the selected volume (35 or 70 μL) with a pipette (Eppendorf® Reference 2, 10–100 μL). For tear collection, the bent tip of a dye-free Schirmer tear strip (Eye Care Product Manufacturing) was placed in the ventrolateral conjunctival fornix of each eye, removed when the 20-mm mark of wetness was reached and transferred to a 2-mL Eppendorf tube (18). In a separate room (to avoid contamination), the dry distal portion of each Schirmer strip was spiked with 5 μL of the internal standard prednisone-d7 (Toronto Research Chemicals, North York, Canada), prepared as 10 ng/μL solution in acetonitrile:water (50:50 v/v). Eppendorf tubes were stored at −80°C until analysis. A combination of centrifugation and solvent elution was used to extract tears from Schirmer strips, using a simplified and optimized method compared to previously reported technique (9, 18, 19): once thawed, each Schirmer strip was placed into a 0.2-mL tube that was pre-punctured at its bottom with an 18-gauge needle, secured into a 2-mL Eppendorf tube via adhesive tape and centrifuged for 2 min at 3,884 g. After the first centrifugation, the strip in the 0.2-mL tube was wetted with 75 μL of methyl tert-butyl ether (MTBE) and allowed to sit for 5 min; of note, this particular solvent was chosen based on its superior ability to extract prednisolone from Schirmer strips as compared to methanol, acetonitrile, and methanol:water 50:50 v/v (pilot experiment using 10 STT per solvent; data not shown). The combination of tubes was centrifuged again at 3,884 g for 2 min. Next, 150 μL acetonitrile was added to each 2-mL tube, vortexed for 30 s, centrifuged at 3,824 g for 1 min, nitrogen dried at 5-15 psi for 10 min, reconstituted with 150 μL acetonitrile:water (25:75 v/v), vortexed for 30 s, centrifuged at 3,884 g for 1 min then transferred to LC-MS vials fitted with glass inserts.

### Systemic Absorption Following Instillation of 1 vs. 2 Drops

Each dog was chosen at random (Excel software) to receive 35 μL or 70 μL of ophthalmic 1% prednisolone acetate suspension in both eyes, four times a day (8 a.m., 12 p.m., 4 p.m., 8 p.m.) for three consecutive days. The course was repeated with each dog receiving the opposite dose following a 48 h washout period, a duration selected on the basis of serum concentrations below limit of quantification at 24 h after topical corticosteroid use in horses (20). On Day 3 of each experiment, blood was collected via peripheral venipuncture 10–15 min following each topical administration, for a total of 4 blood collections per dog and per dose regimen. Blood samples were transferred to EDTA tubes, centrifuged for 30 min at 1,300 g (4°C), and the supernatant plasma was transferred to 2-mL Eppendorf tubes that were stored at −80°C until analysis.

### Drug Quantification in Tears and Plasma Samples

**Figure 1:**
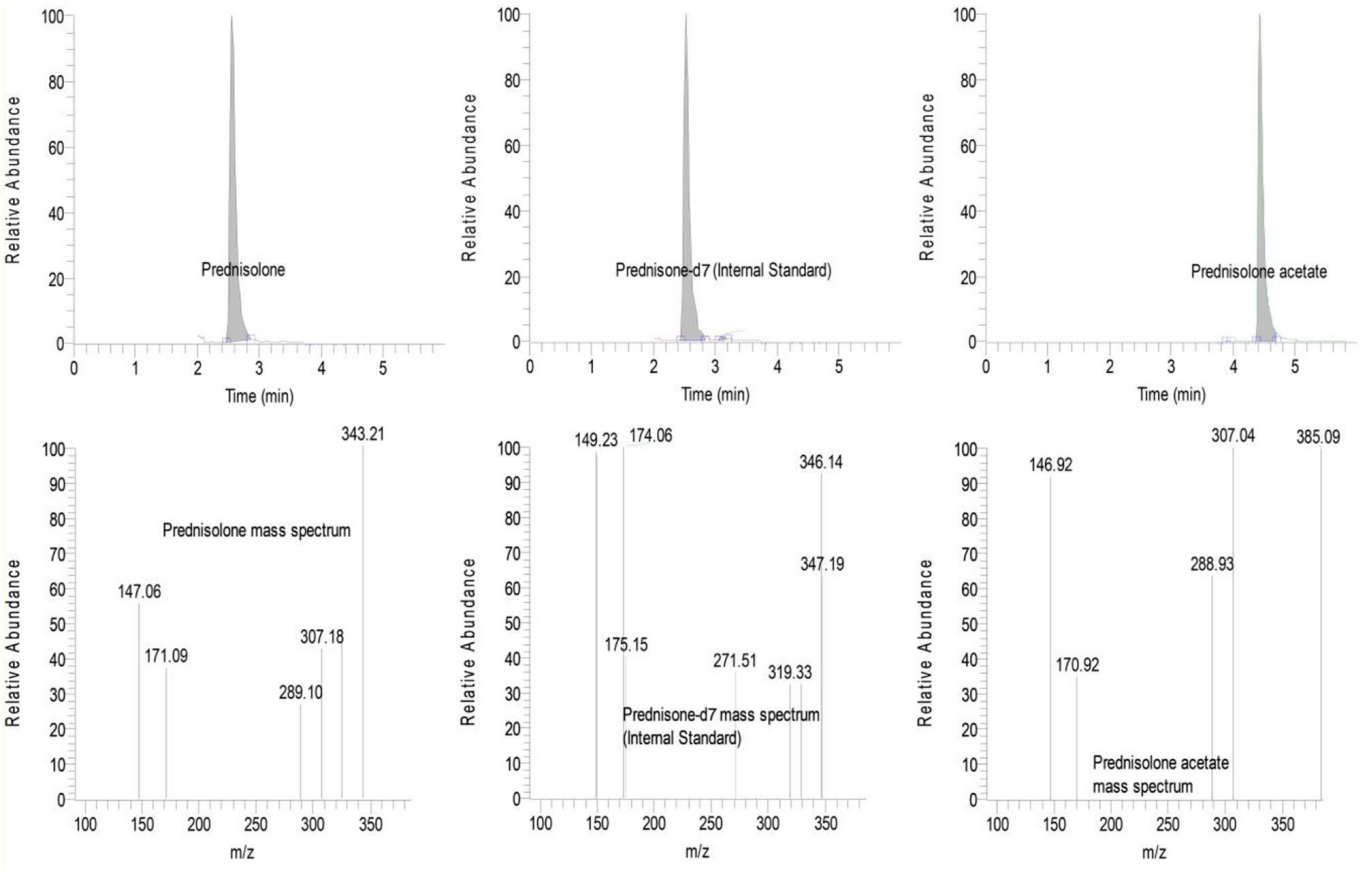
Total ion chromatograms **(Top)** and mass fragmentation patterns **(Bottom)** from a representative tear sample collected 15 min following topical administration of 1% prednisolone acetate in a canine eye. A clear separation is noted in the retention times and/or mass fragmentation profiles of prednisolone **(Left)**, prednisone-d7 internal standard **(Center)** and prednisolone acetate **(Right)**.

Before study initiation, blank canine tears (~15 mL) were collected from the same Beagle dogs using ophthalmic sponges as previously described (21), and blank plasma samples were collected by venipuncture and centrifugation in EDTA tubes. For plasma, 10 standard curve solutions were prepared by spiking blank canine plasma with stock solution of prednisolone and prednisolone acetate (Sigma Chemical, St. Louis, MO, United States) to obtain the following concentrations: 0.5, 1, 2, 5, 10, 20, 50, 100, 200, and 500 ng/mL. For tears, 9 standard curve solutions were prepared by spiking blank canine tears with stock solution of prednisolone acetate to obtain the following concentrations: 0.02, 0.075, 0.2, 0.5, 1.5, 5, 20, 50 and 100 μg/mL. Tear calibration curve samples were processed in a similar manner to biological tear samples, which involved wetting Schirmer strips with standard solution until the 20-mm mark was reached, spiking prednisone-d7 internal standard onto the distal (dry) portion of the strips, and extracting tears with a combination of centrifugation and elution in MTBE (9). Of note, tear samples collected at 0–30 min following topical instillation of 1% prednisolone acetate were diluted with blank tears spiked with prednisone-d7 to ensure the measured concentration would fall within the range of the generated standard curve; specifically, the dilution factors were 1:40 for samples collected at 0 min and 1 min, 1:30 for 3 min and 5 min, and 1:5 for 15 min and 30 min. Three quality control (QC) samples were analyzed at each run (0.05, 0.75 and 10 μg/mL for tears, 3, 15 and 75 ng/mL for plasma). Concentrations of prednisolone and prednisolone acetate in canine tears and plasma were determined using high-pressure liquid chromatography (Surveyor Pump and Autosampler, Thermos Scientific, San Jose, CA, United States), with separation achieved with an ACE Ultracore C18 column (100 mm × 2.1 m, 2.5 μm; Mac Mod Analytical, Chadds Cord, PA, United States) maintained at 50°C, and detection by triple quadrupole mass spectrometry (TSQ Quantum Discovery Max). Injection volume was set to 12.5 μL. The mobile phases consisted of 0.1% formic acid in water (A) and 0.1% formic acid in acetonitrile (B) at a flow rate of 0.25 mL/min. The mobile phase began at 25% B with a linear gradient to 95% B in 6 min, which was maintained for 1 min at 0.35 mL/min, followed by re-equilibration to 25% B at 0.35 mL/min for 2 min. The chromatic peaks for the internal standard, prednisolone (each eluted at 2.77 ± 0.05 min) and prednisolone acetate (eluted at 4.38 ± 0.05 min) were integrated using Xcalibur software (Thermo Scientific, San Jose, CA) (Figure 1). Drug quantitation was based on linear regression analysis of calibration curves (weighted 1/X) using the analyte to internal standard area ratio, resulting in three measures for each sample: prednisolone acetate (prodrug), prednisolone (active metabolite) and prednisolone_total_ (prodrug + active metabolite). Of note, the calibration curve of prednisolone acetate was used for calculation of prednisolone concentrations in tears (as only prednisolone acetate was spiked into the blank tears for standard curve solutions). Therefore, prednisolone concentrations were adjusted by +19% to account for the additional response of prednisolone above that of prednisolone acetate, a correction factor that was calculated as the average response differential between both steroids in mixed solutions varying in concentrations from 50 to 1,000 ng/mL. Drug recovery from Schirmer strips was evaluated by wetting 10 Schirmer strips until 20-mm mark with 100 μg/mL prednisolone acetate, followed by the same extraction and analysis methods used for biological and calibration samples. Calibration curves exhibited a correlation coefficient (*r*^2^) exceeding 0.99 across the concentration range. In tears, the limit of quantitation (LOQ) for both prednisolone and prednisolone acetate was 0.075 μg/mL with a limit of detection (LOD) for both analytes of 0.005 μg/mL. In plasma, the LOQ for both prednisolone and prednisolone acetate was 1.0 ng/mL with an LOD of 0.2 ng/mL.

### Data Analysis

Normality of the data was assessed using the Shapiro-Wilk test. A mixed model for repeated measures (MMRM) was fitted to the data using the R software version 3.6.0 (8). In the model, tear film prednisolone_total_ or prednisolone concentration was the response variable, the group (1 or 2 drops), time (0 to 720 min) and group-by-time interaction were treated as fixed effects, and the animal and animal-by-group interaction were treated as random effects, using animal as block. After the model was fit, the fixed effects were tested, and comparisons between 1 vs. 2 drops were made for the following outcomes: (i) prednisolone_total_ or prednisolone concentrations in tears at each time point, and (ii) percent of prednisolone_total_ remaining at each time point, using the baseline data of 1 drop for both groups in order to account for the different volumes instilled in both eyes. Further, a non-compartmental PK analysis was conducted with PKanalix version 2019R1 (Lixoft, Orsay, France) and the linear-log trapezoidal rule for calculation of the area under the concentration-time curve (AUC_last_); however, since the drug decay over time was non-linear in tears—a finding due to non-constant timing of blinking (mechanism contributing to prednisolone clearance in tears), among others—the analysis cannot determine all PK parameters accurately (e.g., terminal half-life, clearance) and is thereby focused on the following parameters: AUC_last_, average tear film concentration (C_avg_) and maximal tear film concentration (C_max_). Paired *t*-tests were used to compare AUC_last_, C_avg_ and C_max_ of prednisolone_total_ and prednisolone between eyes receiving 1 vs. 2 drops of 1% prednisolone acetate, as well as plasma prednisolone_total_ concentration in dogs receiving 1 drop or 2 drops of 1% prednisolone acetate in both eyes for 3 days, assessing each session separately (8 a.m., 12 p.m., 4 p.m., 8 p.m.) and all 4 sessions combined. Last, the relative absorption of prednisolone_total_ in the systemic circulation was calculated as the ratio of the drug amount administered (700 μg and 1,400 μg for 1 and 2 drops of 1% prednisolone acetate given to both eyes, respectively) by the drug amount present in the blood—determined as the average plasma concentration at steady state multiplied by 815 mL (total blood volume in a 10-kg Beagle dog) (22). Statistical analyses were performed with SigmaPlot 14.0 (Systat software, Point Richmond, CA, United States), and *P* < 0.05 were considered significant.

## Results

Results are presented as mean ± standard deviation (range) as data was normally distributed (*P* > 0.05). Recovery of prednisolone acetate from Schirmer strips was 90.3 ± 7.9 % (79.9–106.8 %).

### Tear Film Concentrations

**Figure 2:**
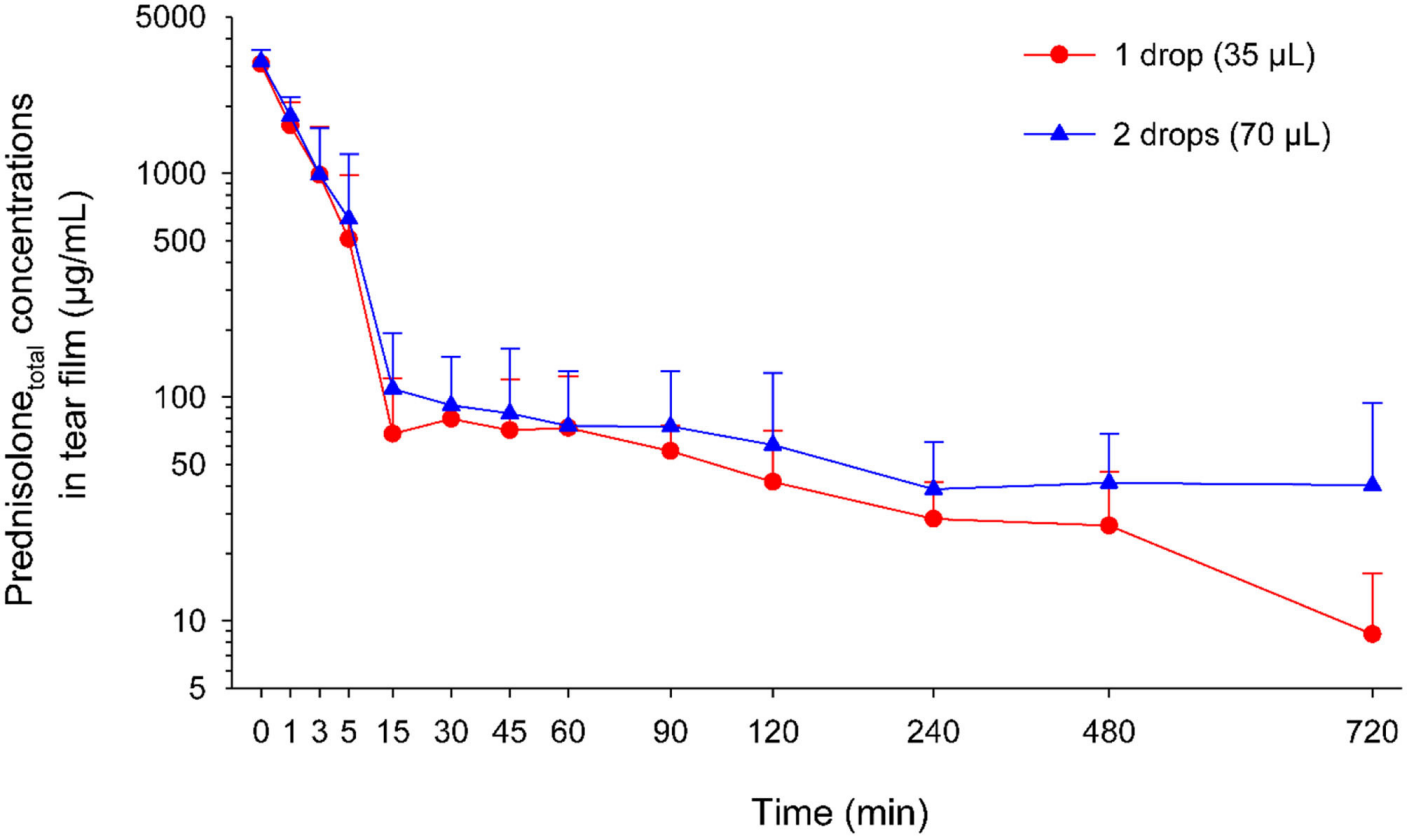
Scatter plot depicting the mean + SD of tear film prednisolone_total_ concentrations over time in canine eyes receiving either one drop (35 μL; red circles) or two drops (70 μL; blue triangles) of 1% prednisolone acetate ophthalmic suspension.

Tear film prednisolone_total_ concentrations following 1 or 2 drops of 1% prednisolone acetate ranged from 1-3786 to 1-3671 μg/mL, respectively (Figure 2), with no statistical differences (*P* ≥ 0.097) between both groups at any time point. Maximal tear film concentration (C_max_) occurred at t = 0 min for eyes receiving 1 drop (3,080 ± 475 μg/mL; range 2,476–3,786 μg/mL) or 2 drops (3,160 ± 404 μg/mL; range 2,314–3,671 μg/mL; *P* = 0.675) of 1% prednisolone acetate. Then, tear film prednisolone concentrations dropped rapidly by ~45% at 1 min and ~95% at 15 min, with no statistical differences (*P* ≥ 0.153) in residual drug levels at any time point between both doses (Table 1). However, the average tear film concentration (C_avg_) and the area under the concentration-time curve (AUC_last_) were significantly greater (*P*= 0.009) for eyes receiving 2 drops (53 ± 17 μg/mL, range 34–87 μg/mL; and 38,331 ± 12,026 min^*^μg/mL; range 24,135–62,358 min^*^μg/mL) compared to 1 drop (38 ± 14 μg/mL, range 14–56 μg/mL; and 27,604 ± 10,002 min^*^μg/mL; range 10,434–40,399 min^*^μg/mL).

**Table 1:** Mean ± SD (range) percent prednisolone_total_ remaining in tears at each time point (1–720 min) compared to *t* = 0 min in canine eyes receiving either 1 drop (35 μL) or 2 drops (70 μL) of 1% prednisolone acetate.

**Time (min)**	**1**	**3**	**5**	**15**	**30**	**45**	**60**	**90**	**120**	**240**	**480**	**720**
**1 drop** **(35** ****μ**L)**	53.5 ± 15.0 (28.4–76.1)	31.6 ± 18.9 (2.5–54.1)	16.9 ± 16.0 (2.3–49.4)	2.2 ± 1.6 (0.6–5.5)	2.5 ± 2.2 (0.3–6.8)	2.2 ± 1.2 (0.9–4.5)	2.5 ± 2.0 (0.4–6.5)	1.9 ± 0.5 (1.0–2.3)	1.3 ± 0.7 (0.5–2.8)	0.9 ± 0.5 (0.4–1.8)	0.9 ± 0.6 (0.2–1.9)	0.3 ± 0.2 (0–0.6)
**2 drops** **(70** ****μ**L)**	57.3 ± 10.6 (39.8–74.0)	31.7 ± 18.7 (4.5–63.4)	19.8 ± 17.3 (1.4–46.4)	3.5 ± 2.7 (0.7–8.2)	3.0 ± 2.0 (0.7–6.5)	2.7 ± 2.5 (0.8–8.6)	2.4 ± 1.9 (0.3–5.8)	2.3 ± 1.8 (1.2–6.7)	1.9 ± 2.1 (0.5–6.5)	1.2 ± 0.8 (0.4–2.6)	1.4 ± 1.2 (0.6–4.2)	1.3 ± 1.8 (0–5.4)
***P*****-value**	0.153	0.483	0.220	0.358	0.450	0.449	0.512	0.449	0.430	0.470	0.440	0.393

*Results are reported in %*.

Tear film prednisolone concentrations following 1 or 2 drops of 1% prednisolone acetate ranged from 0.3–115.7 μg/mL and 0.3–68.6 μg/mL respectively (Table 2), with no statistical differences (*P* ≥ 0.083) between both groups at any time point. On average, the active metabolite prednisolone represented 10% of total drug level over the collection period (0–720 min). Table 3 summarizes the PK parameters for prednisolone_total_ and prednisolone for both doses.

**Table 2:** Mean ± SD (range) tear film prednisolone concentrations at 0–720 min in canine eyes receiving either 1 drop (35 μL) or 2 drops (70 μL) of 1% prednisolone acetate.

**Time (min)**	**0**	**1**	**3**	**5**	**15**	**30**	**45**	**60**	**90**	**120**	**240**	**480**	**720**
**1 drop (35** ****μ**L)**	62.0 ± 25.5 (32.2–115.7)	44.6 ± 16.8 (9.6–62.1)	44.3 ± 13.1 (22.9–63.7)	27.1 ± 9.5 (11.4–41.1)	8.4 ± 6.7 (1.8–20.1)	8.3 ± 6.0 (2.1–18.2)	8.1 ± 3.4 (4.5–13.1)	5.2 ± 2.1 (2.3–8.4)	6.2 ± 4.2 (2.1–15.6)	6.0 ± 2.1 (2.6–8.1)	3.9 ± 2.0 (1.4–7.3)	3.3 ± 2.3 (0.5–6.5)	1.4 ± 1.1 (0.3–2.8)
**2 drops (70** ****μ**L)**	46.4 ± 10.0 (37.2–68.6)	38.2 ± 13.5 (17.6–56.9)	36.9 ± 13.4 (15.8–58.6)	30.6 ± 20.5 (8.6–58.5)	11.4 ± 8.2 (2.5–24.0)	9.8 ± 4.9 (4.1–18.4)	7.7 ± 6.2 (0.8–20.2)	8.8 ± 9.9 (3.1–32.2)	6.1 ± 2.1 (3.4–8.4)	5.6 ± 3.2 (3.0–11.9)	4.8 ± 3.6 (1.5–10.8)	4.1 ± 3.9 (0.3–12.1)	4.0 ± 2.1 (1.7–7.5)
***P*****-value**	0.083	0.487	0.183	0.378	0.463	0.459	0.495	0.449	0.441	0.469	0.459	0.404	0.195

*Results are reported in μg/mL*.

**Table 3:** Mean ± SD (range) tear film pharmacokinetic parameters for prednisolone_total_ and prednisolone in canine eyes receiving either 1 drop (35 μL) or 2 drops (70 μL) of 1% prednisolone acetate ophthalmic suspension: maximal concentration (C_max_), average concentration (C_avg_) and area under the concentration-time curve (AUC_last_).

	**Prednisolone**_****total****_			**Prednisolone**		
	**C_**max**_** **(μg/mL)**	**C_**avg**_** **(μg/mL)**	**AUC_**last**_** (min*μg/mL)	**C_**max**_** **(μg/mL)**	**C_**avg**_** **(μg/mL)**	**AUC_**last**_** (min*μg/mL)
**1 drop** **(35** ****μ**L)**	3,080 ± 475 (2,476–3,786)	38 ± 14 (14–56)	27,604 ± 10,002 (10,434–40,399)	66 ± 24 (32–116)	14 ± 3 (6–17)	2,998 ± 1,112 (1,026–4,342)
**2 drops** **(70** ****μ**L)**	3,160 ± 404 (2,314–3,671)	53 ± 17 (34–87)	38,331 ± 12,026 (24,135–62,358)	50 ± 10 (40–69)	13 ± 4 (7–19)	3,783 ± 1,221 (2,193–5,742)
***P*****-value**	0.675	0.009	0.009	0.135	0.452	0.112

### Plasma Concentrations

At steady state, no significant differences (*P* ≥ 0.113) were noted in plasma prednisolone_total_ concentrations following each of the 4 topical administrations (8 a.m., 12 p.m., 4 p.m., 8 p.m.) whether dogs received 1 drop or 2 drops of 1% prednisolone acetate in each eye. Similarly, mean ± SD plasma prednisolone_total_ concentration for 1 drop (17.0 ± 11.0 ng/mL, range 6.0–50.3 ng/mL) and 2 drops (20.0 ±17.1 ng/mL, range 5.4–93.9 ng/mL) did not differ significantly (*P* = 0.438) when all 4 sessions at Day 3 where combined. Prednisolone concentrations varied from 3.9 to 34.0 ng/mL and represented 72% of total prednisolone concentration in plasma. The relative absorption of topical drug in the blood compartment was 2.0 and 1.2% for dogs receiving 1 or 2 drops of prednisolone acetate in both eyes, respectively.

## Discussion

The present study describes the tear film pharmacokinetics of topical 1% prednisolone acetate (one or two drops) and estimates the fraction of the drug that is absorbed into the systemic circulation of dogs. Of note, the study did not solely report the active metabolite (prednisolone) but instead focused on the overall drug concentration (prednisolone_total_) as it better represents the amount of drug that is available for diffusion into the eye or absorption into the systemic circulation; in fact, the majority of esterase activity that converts prednisolone acetate to prednisolone is located in ocular tissues (e.g., cornea, anterior uvea) and not the tear compartment (17). At first glance, tear film kinetics appeared comparable for eyes receiving 1 or 2 drops of the ophthalmic suspension as there were no statistical differences in the drug concentrations or residual drug levels at each timepoint, and the precorneal retention time was relatively short in both groups (>95% drug loss at 15 min). However, further assessment showed that the average tear film concentration (C_avg_) and the overall drug exposure (AUC_last_) were both significantly higher (by 39%) in eyes receiving two vs. one drop of 1% prednisolone acetate. This finding contrasts with topical instillation of 1% fluorescein solution in dogs (8), and could be partly explained by a larger amount of drug particles being sequestered in the conjunctival cul-de-sac (15, 16) when two drops of an ophthalmic suspension are applied to the ocular surface. Further studies are needed to verify this hypothesis and determine whether this “statistically significant” finding is at all significant from a clinical perspective (i.e., therapeutic benefit, toxicity). The authors suspect differences between 1 vs. 2 drops are likely not relevant in practice as (i) tear film prednisolone levels were > 0.4 ng/mL (10^−9^M) in all dogs throughout the 12-h sampling time, a concentration shown to decrease the expression of deleterious cytokines and matrix metalloproteinases in a rat model of keratitis (23); and (ii) tear film prednisolone levels only exceeded 620 μg/mL for <15 min in both cases, a concentration shown to affect canine corneal epithelial cell morphology and growth *in vitro* when cells are exposed to this level for ≥ 24 h (24).

Further interpretation of tear film PK of 1% prednisolone acetate highlights additional findings of relevance to clinical practice. First, tear prednisolone concentrations at *t* = 0 min (i.e., immediately upon mixing with the tear film) showed an approximately three-fold dilution of the applied drug (average 3.08 mg/mL); this finding is consistent with data from one of our recent studies where we used fluorophotometry and data modeling to estimate a canine tear volume of 65.3 μL (25). Second, when comparing a single drop of 1% prednisolone acetate and oral administration of prednisone (0.5–4.0 mg/kg) (18), tear prednisolone concentrations (1–3,786 vs. 0.005–0.191 μg/mL) and overall drug exposure (AUC_last_, 10,434–40,399 vs. 0.88–50.2 min^*^μg/mL) were much higher with the topical route. Therefore, an eye drop is preferred for treating inflammatory conditions of the avascular cornea. In contrast, the efficacy of both administration routes is likely comparable for diseases of the conjunctiva, based on drug AUCs in conjunctival samples of rabbits (26), although tissue concentrations were not evaluated in the present study. Third, the precorneal retention time of prednisolone ophthalmic suspension was relatively short in the present study, as the initial drug concentration drops by ~45% within 1 min and ~95% within 15 min of topical administration. This short retention time of an ophthalmic suspension is similar to reports of dogs receiving ophthalmic solutions such 1% fusidic acid (10), 0.3% ciprofloxacin (11), and 1% fluorescein (8).

Given the relatively short precorneal retention time of prednisolone suspension, other strategies could be considered to optimize the therapeutic efficacy of topical corticosteroids: (i) Increase the concentration of the formulation—a 1% preparation of prednisolone acetate produced higher drug concentrations in the eye (cornea, conjunctiva, aqueous humor) compared to 0.125 and 0.5% preparations (6, 15); however, 1% is the highest concentration that is commercially available, and further increases in concentration did not augment the therapeutic response in one study of inflammatory keratitis (27); (ii) Increase the viscosity of the formulation—drug concentrations in ocular tissues were higher when prednisolone was coupled with highly viscous polymers such as carboxypolymethylene (28) or carbomer 0.5% (6); (iii) Increase the frequency of administration—reduction in inflammatory cells invading the cornea was significantly better when prednisolone acetate was applied at 1 h compared to 4 h intervals, and was further improved when the drug was applied at 15 min intervals (29); (iv) Consider an alternate corticosteroid—for instance, 0.05% difluprednate administered at a lower frequency than 1% prednisolone acetate is at least as effective in controlling inflammation associated with paracentesis-induced uveitis in dogs (30) or endogenous uveitis in humans (31).

Clinicians need to balance the therapeutic benefits of corticotherapy (and aforementioned strategies to optimize response) with the potential local and systemic adverse effects associated with topical corticosteroid use (1, 2). In fact, cautious use of corticosteroids is always warranted, even when using topical formulations (1, 2). In the present study, 1% prednisolone acetate applied to both eyes four times daily for 3 days resulted in detectable drug levels in the blood. No significant differences were noted in plasma concentrations of dogs receiving 1 vs. 2 drops at each session, reinforcing the fact that the majority of the second drop is not systemically absorbed but is instead wasted by spillage over the periocular skin (8) or absorbed into the eye. Overall, an average of 2.0 and 1.2% of the applied dose (1 and 2 drops in both eyes per session, respectively) reached the systemic circulation–a fraction that is similar to findings in primates (1–7%) (32). It is important to note, however, that these figures represent the minimum (and not total) dose absorbed since they do not account for the drug in the blood cell fraction or drug that diffused to peripheral tissues and other body fluids (e.g., liver, kidneys, bile, urine) (32, 33). Despite this underestimation, the fraction of the dose that is systemically absorbed in dogs is much lower than the typical value reported in several reviews on ocular pharmacology (50–100%) (16, 34–36). This discrepancy can be explained, in part, by a strong focus on the rabbit as the animal selected for basic research in ocular pharmacology; for instance, Sigurdsson et al. showed a 60% systemic absorption of topically applied dexamethasone, a fraction that was comparable to intravenous and intranasal administrations of the same dose in the same rabbits (37). However, the rabbit's eye is poorly representative of humans or companion animals given important differences in anatomy, physiology (e.g., very low blink rate) (38) and tear film dynamics (8, 25).

Plasma prednisolone levels detected in our dogs, albeit relatively small and potentially 50–100-fold lower than other routes of administration (oral, peribulbar or subconjunctival injection) (39–41), are not completely without risk. Topical corticotherapy has been associated with systemic adverse effects in dogs of diverse size and body weight, namely suppression of the hypothalamic-hypophyseal-adrenal axis (up to the development of iatrogenic Cushing's) and hepatic glycogen accumulation (42–45), with similar concerns being reported in human patients (46, 47). Taken together, though topical prednisolone acetate is an excellent drug for inflammatory and immune-mediated diseases of the anterior segment of the eye, caution must be exercised to prevent unwanted and potentially serious adverse effects, regardless of a patient's size. Once inflammation is controlled, frequency of administration should be gradually reduced to find the lowest effective dose and minimize potential toxicity.

The main limitation of the study is the use of dogs from a single breed, all being relatively young and healthy. Tear film PK may differ slightly in smaller or larger canine breeds—presumably due to differences in volume of the tear compartment and conjunctival cul-de-sac (8, 25, 48)—or in older animals, in whom the ocular physiology and tear film dynamics may be altered (49–51). Similarly, patients with ocular diseases may have a different PK profile or systemic absorption following topical corticotherapy, either related to ocular irritation (e.g., reflex tearing) and/or disruption of ocular barriers (e.g., denuded corneal epithelium or breakdown of the blood-tear barrier) (33, 52). In fact, increased systemic absorption is well-documented for drugs administered topically on the inflamed skin (53, 54). Ultimately, the aforementioned factors could be addressed by modeling tears and plasma prednisolone concentrations obtained from a large group of dogs, representing a population of diverse characteristics such as sex, age, and health status (55). Two other limitations may be related to the study's methodology. First, blood sampling was performed at a single timepoint after each topical administration (10–15 min), hence the maximal plasma concentration could have been missed; however, a study on topical dexamethasone in humans undergoing vitrectomy surgery showed a comparable range of plasma concentrations whether blood was collected 3 to 101 min following eye drop administration (41). Second, the exact amount of prednisolone delivered to the eye may have differed from 1 day to another, despite an identical volume administered, as the distribution of drug particles in the ophthalmic suspension is heterogenous (56); however, this drawback was limited by transferring prednisolone suspension into Eppendorf vials that were vigorously shaken for 1 min with the same vortex mixer.

In conclusion, tear film prednisolone concentrations were high (~ 3.1 mg/mL) immediately following topical administration of 1% prednisolone acetate ophthalmic suspension, although drug levels decreased rapidly by ~45% at 1 min and ~95% at 15 min. Instillation of 2 drops vs. 1 drop provided a higher drug exposure to the ocular surface over the 12-h sampling period, although the clinical benefit or toxicity related to the second drop are likely negligible given the relatively low concentrations in tears beyond t = 0 min. Prednisolone acetate reached the systemic circulation at ≤ 2% of the dose applied to the ocular surface, with no differences in plasma levels between 1 and 2 drops applied 4 times daily for 3 days. Judicious use of topical corticotherapy is warranted in a clinical setting to optimize efficacy while minimizing local or systemic toxicity.

## Data Availability Statement

The raw data supporting the conclusions of this article will be made available by the authors, without undue reservation.

## Ethics Statement

The animal study was reviewed and approved by the Institutional Animal Care and Use Committee at Iowa State University.

## Author Contributions

LS conceptualized and designed the study in consultation with JM. LS and NK performed the experiments. LS, LW, and JM analyzed the data. All authors wrote the manuscript.

## Conflict of Interest

The authors declare that the research was conducted in the absence of any commercial or financial relationships that could be construed as a potential conflict of interest.
